# Circulating Interferon-Gamma Levels Are Associated with Low Body Weight in Newly Diagnosed Kenyan Non-Substance Using Tuberculosis Individuals

**DOI:** 10.1155/2016/9415364

**Published:** 2016-01-05

**Authors:** Nathan Shaviya, Valentine Budambula, Mark K. Webale, Tom Were

**Affiliations:** ^1^Department of Medical Laboratory Sciences, Masinde Muliro University of Science and Technology, P.O. Box 190, Kakamega 50100, Kenya; ^2^Department of Environment and Health Sciences, Technical University of Mombasa, Mombasa, Kenya

## Abstract

Although interferon-gamma, interleukin-10, and adiponectin are key immunopathogenesis mediators of tuberculosis, their association with clinical manifestations of early stage disease is inconclusive. We determined interferon-gamma, interleukin-10, and adiponectin levels in clinically and phenotypically well-characterised non-substance using new pulmonary tuberculosis patients (*n* = 13) and controls (*n* = 14) from Kenya. Interferon-gamma levels (*P* < 0.0001) and interferon-gamma to interleukin-10 (*P* < 0.001) and interferon-gamma to adiponectin (*P* = 0.027) ratios were elevated in tuberculosis cases. Correlation analyses in tuberculosis cases showed associations of interferon-gamma levels with body weight (*ρ* = −0.849; *P* < 0.0001), body mass index (*ρ* = 0.664; *P* = 0.013), hip girth (*ρ* = −0.579; *P* = 0.038), and plateletcrit (*ρ* = 0.605; *P* = 0.028); interferon-gamma to interleukin-10 ratio with diastolic pressure (*ρ* = −0.729; *P* = 0.005); and interferon-gamma to adiponectin ratio with body weight (*ρ* = −0.560; *P* = 0.047), body mass index (*ρ* = −0.604; *P* = 0.029), and plateletcrit (*ρ* = 0.793; *P* = 0.001). Taken together, our results suggest mild-inflammation in early stage infection characterised by upregulation of circulating interferon-gamma production in newly infected TB patients.

## 1. Introduction

Tuberculosis (TB) due to* Mycobacterium tuberculosis* is an important cause of infectious disease burden in the world. An estimated 9.0 million cases, 5.7 million new cases, and 1.5 million deaths were attributable to TB in 2013 [1]. A vast majority of new TB cases occur in developing countries particularly among individuals living in crowded and informal settlements and those with underlying conditions such as HIV, diabetes, malnutrition, smoking, and alcohol abuse [2]. Sub-Saharan Africa accounts for the highest burden of TB comprising 2.8 million cases including 2.3 million new cases and 230,000 deaths [1]. Kenya is among the high TB burdened countries with annual incidence of 110,000 cases, 90,000 new infections, and over 4,000 deaths [1].

While molecular mechanisms underlying TB-related burden are poorly understood, host inflammatory responses mediate pathogenesis of the disease [3]. Previous studies showed elevated plasma IFN-*γ* with lower IL-10 levels in first-time TB smear positive patients [4]. In contrast, increased plasma IL-10 levels in the presence of lower concentrations of IFN-*γ* were detected in chronic TB patients [5]. In addition, previous studies showed a higher IFN-*γ* to IL-10 ratio in treatment-naive newly infected TB patients [6, 7], suggesting that* M. tuberculosis* infections are characterised by an early burst in the proinflammatory cytokine response followed by an increased anti-inflammatory cytokine response in the chronic phases of disease.

The inflammatory cytokine response is linked to development of clinical manifestations of TB. For instance, higher levels of IFN-*γ* and IL-10 are associated with lower body weight and wasting in newly infected TB patients [8, 9]. Whilst a higher IFN-*γ* to IL-10 ratio is associated with protection and TB cure [10], a lower IFN-*γ* to IL-10 ratio is linked to TB relapse [6, 11]. Furthermore, increased plasma adiponectin levels are associated with severe TB characterised by extensive pulmonary lesions [12]. Although adiponectin promotes IL-10 release and impairs IFN-*γ* secretion in human macrophages [13], the magnitude of IFN-*γ* and IL-10 to adiponectin production as an indicator of inflammatory response and clinical manifestations in TB is largely unknown.

Increasing evidence indicate that host response to TB is likely to be compounded by underlying patient factors including malnutrition, coinfections, and alcohol and substance consumption. For example, plasma levels of IFN-*γ* increase in acute HIV infection [14] and decrease in chronic HIV infection [15], suggesting that stage of HIV infection determines inflammatory cytokine responses. In addition, circulating adiponectin levels are decreased as IFN-*γ* and IL-10 levels are elevated in individuals with malnutrition and obesity [16–19], indicating that malnutrition promotes alterations in inflammatory cytokine production. Substance use also influences adiponectin, IL-10, and IFN-*γ* production. For instance, marijuana components induce IFN-*γ* and suppress IL-10 production [20, 21] while alcohol increases IFN-*γ* and IL-10 levels [22]. In addition, reduced IFN-*γ* and IL-10 levels were found in opiate addicts [23] while low levels of adiponectin were reported in cocaine, opiate, cigarette, and alcohol users [24–27]. Taken together, underlying disease conditions and substance consumption are key factors promoting increased dysregulation in the inflammatory response in TB [28, 29]. To our knowledge, however, no study has examined cytokine levels and their clinical correlates in phenotypically and clinically well-characterised newly infected pulmonary TB patients. As such, the present study determined circulating IFN-*γ*, IL-10, and adiponectin levels and their association with clinical manifestations of early stage disease in non-substance using newly diagnosed pulmonary TB cases.

## 2. Materials and Methods

### 2.1. Study Design and Participants

This cross-sectional study was conducted among consenting new pulmonary TB patients and controls at Bomu Hospital, a social enterprise facility in Mombasa, a coastal city in Kenya. Substance and drug using individuals [30] and those presenting with underlying conditions such as HIV and diabetes including retreatment TB cases were excluded from the study. Newly diagnosed pulmonary TB patients were defined as individuals with a first case of TB positive sputum smear while controls comprised individuals with TB negative sputum smears and presenting with no evidence of illness. A total of 13 newly diagnosed pulmonary TB cases and 14 controls were recruited in this study. The sample size was calculated based on the formula of [31] and plasma IFN-*γ* concentrations previously determined in TB patients and controls [32]. Chest X-rays were taken on all study participants on the day of TB diagnosis and independently interpreted by two radiologists.

### 2.2. Physical Measurements

Anthropometric measures of the study participants were obtained by trained clinicians. Body weight was measured to the nearest 0.1 kg in light clothes, and height was measured to the nearest 1.0 cm in an upright posture. Waist circumference (WC) was assessed to the nearest 0.1 cm at smallest diameter between the iliac crest and lower rib. Hip circumference (HC) was measured to the nearest 0.1 cm around the maximum circumference of the buttocks. Middle upper arm circumference (MUAC) was measured midway to nearest 0.1 cm amid the tip of acromion and olecranon process. Body mass index (BMI, kg/m^2^) was calculated as weight (kg)/height (m) while waist-to-hip ratio was calculated as WC (cm)/HC (cm).

### 2.3. Vital Signs

Axillary temperature was obtained using a digital thermometer. Systolic and diastolic blood pressures were measured to the nearest 2 mmHg after a 10-minute rest in a sitting position using a digital blood pressure machine. Pulse rate was determined after a 10-minute rest in the sitting position using fingertip heart rate monitor.

### 2.4. Laboratory Diagnosis of TB

Sputum was obtained at enrolment and subsequently the following morning from all consenting individuals and used for smear preparation and acid-fast (Ziehl-Neelsen) staining. Smears were examined for presence of* M. tuberculosis* and bacilli were enumerated and scored according to the global guidelines for laboratory diagnosis of TB [33].

### 2.5. Haematological Enumerations

About 3.0 mL venous blood was collected from the study participants in ethylenediaminetetraacetic acid Vacutainer tubes (Becton Dickinson, Franklin Lakes, USA). Complete blood count was performed using BC-3200 Mindray autohaematology analyser (Mindray Inc., Mahwah, USA). The system was calibrated every morning before sample analysis, and hematologic analyses were performed within 10 minutes of blood collection.

### 2.6. Plasma Preparation

Plasma samples were prepared by centrifugation using bench-model centrifuge (Forma Scientific, Inc., Ohio, USA). Briefly, blood was centrifuged for 10 minutes at 1500 ×g, aliquoted into labelled cryovials, and frozen at −80°C until use for cytokine measurements.

### 2.7. Cytokine Measurements

Circulating levels of IFN-*γ*, IL-10, and adiponectin were determined in plasma samples using a sandwich enzyme linked immunosorbent assay (ELISA) according to the manufacturer's protocols (R&D Systems, Inc., Minneapolis, USA).

### 2.8. Statistical Analysis

Data analysis was conducted using GraphPad Prism v5 (GraphPad Inc., California, USA). Comparisons in age, anthropometric and hematologic measures, and cytokine levels between cases and controls were performed using Mann-Whitney *U* test. Fisher's exact test was used for comparing gender distribution between the study groups. Spearman's rank correlation test was used to examine associations of cytokine levels and cytokine ratios with anthropometric and clinical measures in the TB cases.

### 2.9. Ethical Considerations

This study was approved by Kenyatta University Ethical Review Committee and was conducted according to Helsinki's declarations. Written informed consent was obtained from all study participants prior to enrolment into the study. TB cases were treated according to Kenya national guidelines for TB treatment that is consisted of isoniazid [34].

## 3. Results

### 3.1. Baseline Characteristics of the Study Participants

Baseline demographic and clinical information of the study participants are shown in Table 1. Age (*P* = 0.126) and gender distribution (*P* = 0.695) were similar between the study groups. Anthropometric assessment showed significantly lower body weight (*P* = 0.028), body mass index (*P* = 0.024), hip circumference (*P* = 0.032), and middle upper arm circumference (*P* < 0.0001) in cases relative to controls. However, height (*P* = 0.145), waist girth (*P* = 0.120), and waist-to-hip ratio (*P* = 0.846) were similar between the study groups. Among the presenting clinical manifestations, axillary body temperature (*P* = 0.006) was significantly elevated with lower systolic (*P* = 0.004) and diastolic (*P* = 0.002) pressures in the cases. However, pulse rate (*P* = 0.744) was similar between the two groups. Chest examination indicated that 77.0% of the TB cases had congestion (30.8%) or crepitation (46.2%). Hematologic analyses showed similar levels of total leucocyte (*P* = 0.120), neutrophil (*P* = 0.627), lymphocyte (*P* = 0.980), monocyte (*P* = 0.209), eosinophil (*P* = 0.112), and platelet (*P* = 0.961) counts. Interestingly, TB cases presented with lower haemoglobin (*P* = 0.032), mean platelet volume (*P* = 0.001), and plateletcrit (*P* = 0.025).

### 3.2. Plasma Cytokine Levels

**Figure 1:**
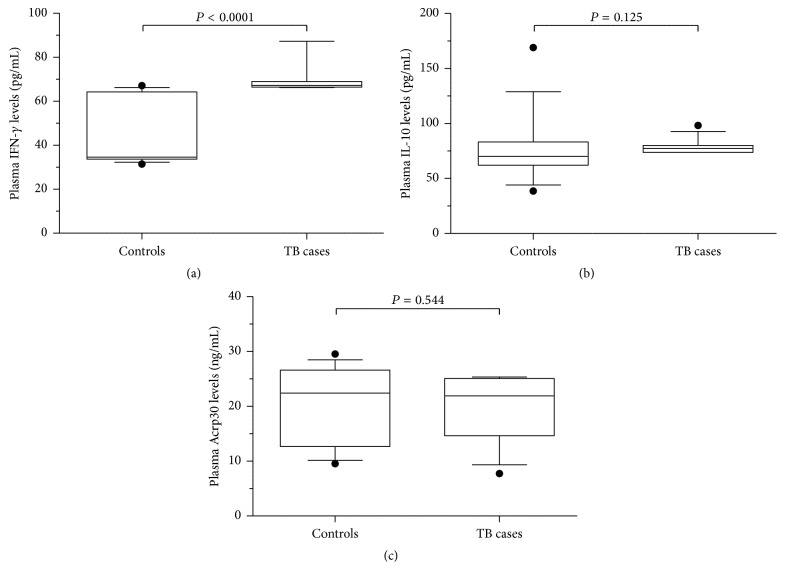
Circulating cytokine levels in new tuberculosis (TB) cases (*n* = 13) and controls (*n* = 14). (a) Circulating interferon-gamma (IFN-*γ*) levels in TB cases and controls. (b) Circulating interleukin-10 (IL-10) levels in TB cases and controls. (c) Circulating adiponectin (Acrp30) levels in TB cases and controls. Data are presented as box plots, where the box represents the interquartile range, the line through the box represents the median, whiskers indicate the 10th and 90th percentiles, and the closed circles represent outliers. Statistical comparisons were performed using the Mann-Whitney *U* test. IFN-*γ* levels (pg/mL) were elevated in TB cases compared to controls (*P* < 0.0001). IL-10 (pg/mL; *P* = 0.125) and adiponectin (ng/mL; *P* = 0.544) levels were similar between the TB cases and controls.

**Figure 2:**
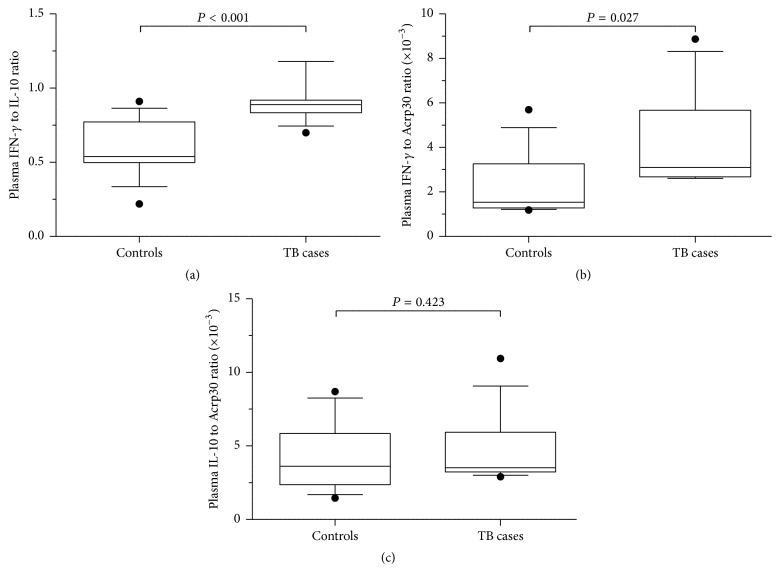
Circulating cytokine ratios in new tuberculosis (TB) cases (*n* = 13) and controls (*n* = 14). (a) Circulating interferon-gamma (IFN-*γ*) to interleukin-10 (IL-10) ratio in TB cases and controls. (b) Circulating IFN-*γ* to adiponectin (Acrp30) ratio in new TB cases and controls. (c) Circulating IL-10 to adiponectin (Acrp30) ratio in new TB cases and controls. Data are presented as box plots, where the box represents the interquartile range, the line through the box represents the median, whiskers indicate the 10th and 90th percentiles, and the closed circles represent outliers. Statistical comparisons were performed using the Mann-Whitney *U* test. IFN-*γ* to IL-10 ratios were higher in the TB cases relative to controls (*P* < 0.001). IFN-*γ* to adiponectin ratios were also increased in TB cases relative to controls (*P* = 0.027). IL-10 to adiponectin ratios were similar in TB cases relative to controls (*P* = 0.423).

Plasma levels of IFN-*γ* were significantly elevated in TB cases (median, 67.2 pg/mL; IQR, 2.5 pg/mL) compared to controls (median, 34.7 pg/mL; IQR, 30.5 pg/mL; *P* < 0.0001; Figure 1(a)). Plasma IL-10 (median, 77.2 pg/mL; IQR, 6.4 pg/mL versus 70.0 pg/mL; IQR, 20.8; *P* = 0.125; Figure 1(b)) and adiponectin (median, 21.9 ng/mL; IQR, 10.4 ng/mL versus 22.4 pg/mL; IQR, 13.9 pg/mL; *P* = 0.544; Figure 1(c)) levels were, however, similar between cases and controls. In addition, ratios of IFN-*γ* to IL-10 (median, 0.9; IQR, 0.1; *P* < 0.001 versus median, 0.5; IQR, 0.3; Figure 2(a)) and IFN-*γ* to adiponectin (median, 3.1 × 10^−3^; IQR, 3.0 × 10^−3^ versus median, 1.5 × 10^−3^; IQR, 2.0 × 10^−3^; *P* = 0.027; Figure 2(b)) were significantly higher in cases relative to controls. IL-10 to adiponectin ratio was similar in cases (median, 3.5 × 10^−3^; IQR, 2.7 × 10^−3^) and controls (median, 3.6 × 10^−3^; IQR, 3.5 × 10^−3^; *P* = 0.423; Figure 2(c)).

### 3.3. Associations of Cytokines with Physical and Clinical Measures

Associations of cytokines with physical and clinical measures are shown in Table 2. Plasma IFN-*γ* levels inversely correlated with body weight (*ρ* = −0.849; *P* < 0.0001), BMI (*ρ* = 0.664; *P* = 0.013), and hip circumference (*ρ* = −0.579; *P* = 0.038) and positively correlated with plateletcrit (*ρ* = 0.605; *P* = 0.028). In addition, IFN-*γ* to IL-10 ratio inversely correlated with diastolic pressure (*ρ* = −0.729; *P* = 0.005) while IFN-*γ* to adiponectin ratio inversely correlated with weight (*ρ* = −0.560; *P* = 0.047) and BMI (*ρ* = −0.604; *P* = 0.029) and positively correlated with plateletcrit (*ρ* = 0.793; *P* = 0.001).

## 4. Discussion

*Mycobacterium tuberculosis* infections evolve through an early stage and subsequently a chronic course of disease. Proinflammatory cytokines such as IFN-*γ* are upregulated in the early phase followed by counteractive increase in anti-inflammatory cytokines such as IL-10 and adiponectin in the chronic phase of disease [3]. Since the magnitude and timing of the cytokine response in TB are influenced by underlying host factors [28, 29], to our knowledge, this is the first study to examine circulating IFN-*γ*, IL-10, and adiponectin levels and their relationship with clinical manifestations of early phase infection in a cohort of non-substance using newly TB infected patients.

The elevated circulating IFN-*γ* levels in the TB cases suggest upregulation in the proinflammatory response. These results are consistent with previous studies showing elevated plasma levels of IFN-*γ* in newly infected TB patients [35–37]. The fact that IFN-*γ* levels are upregulated in the newly infected TB cases indicates innate protective response during early phase of* M. tuberculosis* infection. This hypothesis is supported by previous studies showing correlation of IFN-*γ* production and hastened recovery from TB [37] while IFN-*γ* receptor deficiency is associated with increased susceptibility and development of severe disease in* M. tuberculosis *infection [38, 39]. Although the mechanisms underlying increased IFN-*γ* production were not examined in the present study, uptake of* M. tuberculosis* bacilli triggers release of proinflammatory mediators and promotes antimycobacterial effects of alveolar macrophages and dendritic cells during early stage disease [40]. However, hyperactivation of macrophages and dendritic cells leads to overproduction of proinflammatory mediators that mediate development of clinical manifestations in early stage disease [3]. It is, therefore, possible that the upregulation of the proinflammatory response in early phase of* M. tuberculosis* infection is capable of controlling TB infection.

Consistent with elevated IFN-*γ* levels, higher IFN-*γ* to IL-10 and IFN-*γ* to adiponectin ratios were observed in the TB cases. These findings are, in part, consistent with previous studies showing higher IFN-*γ* to IL-10 ratio among antitubercular drug sensitive mycobacteria infected patients and newly diagnosed HIV and TB coinfected individuals [7, 11]. Since host immune responses are dependent on the bacterial load [41], we theorize that the higher ratios of IFN-*γ* to IL-10 and IFN-*γ* to adiponectin mirror upregulation of proinflammatory cytokine response in early stage mycobacterial infection. This proposition is supported by previous studies reporting that higher IFN-*γ* to IL-10 ratio is associated with hyperinflammation leading to disease severity in newly diagnosed HIV and TB coinfected patients [7, 10]. In addition, adiponectin inhibits bacterial lipopolysaccharide-induced production of proinflammatory cytokines suppressing macrophage mycobactericidal effects [42]. Therefore, it appears that IFN-*γ* to IL-10 and IFN-*γ* to adiponectin ratios are good correlates of inflammatory cytokine dysregulation during early stage TB in adults.

The indirect link of IFN-*γ* levels and body weight, body mass index, and hip girth in the TB cases may indicate that IFN-*γ* promotes weight loss in newly infected TB patients. These findings corroborate previous studies showing higher levels of IFN-*γ* in TB cases presenting with lower body weight [8]. Although the mechanisms underlying association between IFN-*γ* levels and nutrition status were not examined in the present study, it is possible that IFN-*γ* contributes to weight loss in newly infected TB patients. For instance, increased IFN-*γ* levels induces anorexia and upregulates IL-12 promoting weight loss through epithelial cell damage [43, 44]. In addition, malnutrition, a risk factor for TB, is prevalent in Kenya and is associated with higher risk of presenting with TB at hospital [45] and increased production of proinflammatory cytokines including IFN-*γ* in TB patients [8, 19]. Furthermore, the inverse link of IFN-*γ* to adiponectin ratio with body weight and body mass index supports a link between low fat store and underlying inflammation in pulmonary TB patients. These results are, in part, consistent with previous studies showing negative correlation between adiponectin levels and body mass index in asymptomatic TB patients [12]. Thus, higher IFN-*γ* relative to adiponectin production may be a useful surrogate indicator of weight loss in newly infected pulmonary TB patients.

The inverse correlation of IFN-*γ* to IL-10 ratio and diastolic pressure in TB patients suggests that the relative balance of the pro- and anti-inflammatory cytokines determines development of TB-associated cardiopathologies. A notable finding in our study supporting this hypothesis is the lower systolic and diastolic pressures and higher rates of chest congestion or crepitation in the TB cases. These results partly mirror previous studies showing lower systolic and diastolic blood pressures in TB patients presenting with heart failure [46]. The fact that reduced diastolic and systolic pressures correlate with TB-related cardiopathological manifestations is further supported by previous clinical studies showing that diastolic dysfunction and echocardiographic grading mirror TB severity [46]. In addition, most TB patients present with adrenal insufficiency that is linked to destruction of the adrenal gland leading to lower systolic and diastolic blood pressures [47–49]. Therefore, our results suggest that IFN-*γ* to IL-10 ratio is a useful indicator of TB-associated cardio- and adrenopathologies.

The positive relationships of IFN-*γ* levels and IFN-*γ* to adiponectin ratio with plateletcrit reflect low-grade inflammation in new TB cases. In support of this interpretation, the plateletcrit and mean platelet volume were lower in the TB cases. Our findings are partly consistent with previous studies showing that lower plateletcrit correlates with mild TB [50], while increased plateletcrit is associated with thrombocytosis and acute phase reactants in severe TB patients [51]. Likewise, increased mean platelet volume parallels radiological extent of disease in active TB patients [52] as reduced haemoglobin correlates with C-reactive protein and erythrocyte sedimentation rate in newly diagnosed and active pulmonary TB patients [51, 53]. Therefore, underlying inflammation may be linked to alterations in the production of IFN-*γ*, adiponectin, plateletcrit, mean platelet volume, and haemoglobin in newly infected non-substance using TB patients.

It is important to emphasize the limitations of the present study. The findings of the present study must be taken with caution because of the small sample size and the cross-sectional design. Although a prospective design would be valuable in providing insights into the inflammatory response in newly infected TB patients, our cross-sectional study provides baseline information on the association between cytokine response and clinical manifestations of TB during early stage disease in Kenya. TB incubation period varies within patients [54]; therefore, it was not possible to determine the duration of infection in these patients. Even though mycobacterial-recall positive individuals produce higher levels of proinflammatory cytokines [55], elevated IFN-*γ* levels in the newly infected TB cases are largely due to activation of innate response to* M. tuberculosis* infection as BCG-vaccines induce long-term memory CD4+ and CD8+ T cell responses [56]. Consequently, analysing circulating IFN-*γ* levels in prospective cohort of newly infected TB patients will confirm the diagnostic value of higher (values above the median level) versus lower (values below the median level) IFN-*γ* production. Furthermore, flow cytometric and cellular analyses and* in vitro* stimulation of peripheral blood and alveolar lavage mononuclear cells from TB patients will identify key inflammatory mediators that are dysregulated and predict development of clinical manifestations of disease.

This study, therefore, provides evidence for the status and association of IFN-*γ* levels, IL-10, and adiponectin levels with clinical manifestations of TB in a well-characterised cohort of non-substance using newly infected TB patients. Our results suggest that IFN-*γ* levels and the ratios of IFN-*γ* to IL-10 and IFN-*γ* to adiponectin are elevated in non-substance using newly infected TB patients. Furthermore, IFN-*γ* levels and the ratios of IFN-*γ* to IL-10 and IFN-*γ* to adiponectin are associated with low body weight, body mass index, hip girth, diastolic pressure, and plateletcrit, respectively, suggesting mild-inflammation during early stage infection in non-substance using newly infected TB patients.

## Figures and Tables

**Table 1:** Baseline characteristics of the study participants.

Characteristic	Controls, *n* = 14	TB cases, *n* = 13	*P* values
Age, yrs.	26.1 (9.4)	33.0 (9.9)	0.126
Females, *n* (%)	6 (42.9)	4 (30.8)	0.695
Height, m	1.7 (0.2)	1.7 (0.1)	0.145
Weight, kg	65.5 (15.5)	56.0 (7.0)	**0.028**
Body mass index, kg/m^2^	23.1 (6.6)	18.7 (3.5)	**0.024**
Waist circumference, cm	86.3 (13.8)	82.0 (13.5)	0.120
Hip circumference, cm	100.5 (9.8)	87.0 (21.0)	**0.032**
Waist-to-hip ratio	0.9 (0.1)	0.9 (0.1)	0.846
MUAC, cm	28.0 (6.5)	22.0 (5.5)	<**0.0001**
Axillary temperature, °C	36.0 (0.3)	36.6 (2.1)	**0.006**
Systolic pressure, mmHg	125.0 (10.0)	110.0 (20.0)	**0.004**
Diastolic pressure, mmHg	80.0 (13.0)	70.0 (10.0)	**0.002**
Pulse rate, bpm	82.0 (5.5)	80.0 (2.0)	0.744
Chest X-ray, *n* (%)			
Congested	0 (0.0)	4 (30.8)	—
Crepitation	0 (0.0)	6 (46.2)	
Normal	14 (100.0)	3 (23.1)	
Leucocytes × 10^3^/*μ*L	6.4 (4.0)	4.3 (2.0)	0.120
Neutrophils × 10^3^/*μ*L	2.1 (3.1)	2.2 (1.6)	0.627
Lymphocytes × 10^3^/*μ*L	1.6 (0.8)	1.5 (0.7)	0.980
Monocytes × 10^3^/*μ*L	0.3 (1.0)	0.2 (0.2)	0.209
Eosinophils × 10^3^/*μ*L	0.2 (0.2)	0.1 (0.1)	0.112
Haemoglobin, g/dL	14.3 (1.4)	12.5 (1.9)	**0.032**
Platelets × 10^9^/*μ*L	336 (116)	358 (255)	0.961
Mean platelet volume, fL	0.2 (0.0)	0.1 (0.1)	**0.001**
Plateletcrit, %	5.8 (2.1)	5.0 (1.3)	**0.025**

Data are presented as medians (IQR, interquartile range) or as indicated. Statistical analysis was performed using Mann-Whitney *U* test for continuous measures and Fisher's exact test for gender distribution. TB, tuberculosis. MUAC, middle upper arm circumference. Values in bold are significant *P* values.

**Table 2:** Correlation of cytokines with physical and clinical measures.

Characteristic	IFN-*γ*		IFN-*γ* to IL-10 ratio		IFN-*γ* to Acrp30 ratio	
	*ρ*	*P*	*ρ*	*P*	*ρ*	*P*
Body weight, kg	**−0.849**	**<0.0001**	−0.126	0.683	**−0.560**	**0.047**
Body mass index, kg/m^2^	**−0.664**	**0.013**	0.126	0.683	**−0.604**	**0.029**
Hip circumference, cm	**−0.579**	**0.038**	−0.132	0.667	−0.334	0.264
MUAC, cm	−0.006	0.985	0.046	0.881	0.046	0.882
Axillary temperature, °C	0.494	0.086	0.011	0.971	0.525	0.066
Systolic pressure, mmHg	−0.373	0.209	−0.369	0.215	−0.189	0.537
Diastolic pressure, mmHg	0.322	0.283	**−0.729**	**0.005**	0.344	0.249
Haemoglobin, g/dL	−0.215	0.481	−0.400	0.176	0.133	0.665
Mean platelet volume, fL	−0.360	0.227	−0.134	0.662	−0.492	0.088
Plateletcrit, %	**0.605**	**0.028**	0.056	0.857	**0.793**	**0.001**

Data presented are correlation coefficient (rho, *ρ*) with associated *P* values. Statistical analysis was performed using Spearman's rank correlation test. IFN-*γ*, interferon-gamma. IL-10, interleukin-10. Acrp30, adiponectin. MUAC, middle upper arm circumference. Values in bold indicate significant *P* values.
